# Endoscopy-assisted anterior cervical debridement combined with posterior fixation and fusion for the treatment of upper cervical spine tuberculosis: a retrospective feasibility study

**DOI:** 10.1186/s12891-022-05084-4

**Published:** 2022-02-08

**Authors:** Zheng Liu, Zhenchao Xu, Yilu Zhang, Xiyang Wang, Zhen Zhang, Dingyu Jiang, Runze Jia

**Affiliations:** 1 Hunan Children’s Hospital, 86# Ziyuan Road, Changsha, 410007 Hunan China; 2 Department of Spine Surgery and Orthopaedics, Xiangya Hospital of Central South University, 87# Xiangya Road, Changsha, 410008 Hunan China; 3 Hunan Engineering Laboratory of Advanced Artificial Osteo-Materials, 87# Xiangya Road, Changsha, 410008 Hunan China

**Keywords:** Upper cervical spine, Tuberculosis, Endoscopy, Anterior cervical debridement, Posterior fixation, Posterior fusion

## Abstract

**Background:**

This retrospective study aimed to determine the feasibility and efficacy of endoscopy-assisted anterior cervical debridement combined with posterior fixation and fusion in patients with upper cervical spine tuberculosis.

**Methods:**

Between June 2008 and January 2016, 17 patients (10 men and 7 women) with upper cervical spine tuberculosis underwent endoscopy-assisted anterior cervical debridement combined with posterior fixation and fusion. Anti-tuberculosis treatment was administered for 2–4 weeks preoperatively and 12–18 months postoperatively. The clinical and radiographic data of the patients were analyzed.

**Results:**

The operation was successfully completed in all patients. Neck pain and stiffness were relieved after the surgery in all patients. The mean operation time was 210.0 ± 21.2 min, and the mean intraoperative blood loss was 364.7 ± 49.6 mL. The mean follow-up duration was 68.1 ± 6.7 months. The erythrocyte sedimentation rate returned to normal by 3 months postoperatively. Visual analog scale scores for neck pain were significantly lower postoperatively than preoperatively. All patients had significant postoperative neurological improvement. Patient-reported outcomes, as measured using the Kirkaldy-Willis criteria, were as follows: excellent, 12 patients; good, 4 patients; fair, 1 patient; and poor, 0 patients. Bone fusion was achieved at 10.9 ± 1.9 months after the surgery; no cases of instrument loosening or fracture occurred.

**Conclusion:**

Endoscopy-assisted anterior cervical debridement combined with posterior fixation and fusion is a feasible and effective surgical method for the treatment of upper cervical spine tuberculosis. It can be used to restore upper cervical spine stability and facilitate spinal healing.

## Background

Upper cervical spine tuberculosis (TB) is a rare infectious disease, which accounts for only 0.3 to 1% of all cases of spinal TB [1–3]. The upper cervical spine is the most mobile and most complex part of the cervical spine. Because of the relatively wide spinal canal and strong tolerance to spinal cord compression in this section of the spine, the early clinical symptoms of upper cervical spine TB are relatively silent and tend to go unnoticed by patients. By the time the patient seeks treatment, extensive bone and soft-tissue destruction have usually occurred, resulting in spinal cord compression and upper cervical spine instability, which can seriously damage the medulla and spinal cord, and cause paralysis, bulbar paralysis, and respiratory dysfunction [4–6]. Therefore, the window of opportunity for conservative treatment is often missed, and the deep position of the infection and the severe damage to the atlantoaxial lateral mass, vertebral body, and pedicle usually necessitate surgical intervention [7].

Currently, transoral debridement combined with fixation via the posterior approach is used to clear the lesion and stabilize the upper cervical spine [8]. However, this procedure carries a certain risk of associated bacterial contamination through the oral cavity. Furthermore, prolonged intubation or tracheotomy, and postoperative enteral tube feeding are unavoidable [9]. It has been reported that debridement via the anterior cervical and retropharyngeal approach combined with posterior fixation and fusion can achieve desirable results [10]. However, it is difficult to operate under direct vision owing to the anatomical complexity of the upper cervical spine. An endoscopic approach may be used to resolve this problem.

This retrospective study aimed to determine the feasibility and efficacy of endoscopic anterior cervical debridement combined with posterior fixation and fusion for the treatment of patients with upper cervical spine TB.

### Study design and ethics statement

This study was a retrospective review of the medical data of 17 patients with upper cervical spine TB who underwent endoscopy-assisted anterior cervical debridement with posterior fixation and fusion at our Hospital, between June 2008 and January 2016.

### Patient selection criteria

The inclusion criteria for this study were as follows: (i) persistent neck pain and stiffness caused by cervical spinal instability, (ii) progressive spinal cord compression, (iii) unpreventable progressive atlantoaxial dislocation, and (iv) lack of lesion absorption despite anti-TB treatment. The exclusion criteria were as follows: (i) intolerance to surgery as determined by preoperative evaluations, (ii) effective conservative treatment, and (iii) patient’s inability to behave autonomously, rendering complete follow-up impossible.

### Clinical evaluations and diagnosis

All patients reported having non-specific symptoms, such as neck pain and stiffness, local tenderness, moderate fever, sweats, weight loss, general weakness, and neurological dysfunction. No patient was HIV positive or had active pulmonary TB. In all patients, upper cervical spine TB was diagnosed on the basis of the clinical presentation, results of laboratory tests, and findings of imaging examinations, such as spinal radiography, computed tomography (CT), and magnetic resonance imaging (MRI). Neck pain was evaluated using the visual analogue scale (VAS), and neurological deficit was assessed using the American Spinal Injury Association (ASIA) impairment scale and the Japanese Orthopaedic Association (JOA) score.

### Preoperative preparations

At least 2 weeks before the surgery, all patients received treatment with the following anti-TB drugs: 300 mg/day isoniazid, 450 mg/day rifampicin, 750 mg/day ethambutol, and 750 mg/day pyrazinamide. Nutrition support is essential, and was offered to all patients. Halo traction was used preoperatively to partially correct atlantoaxial subluxation. In addition, bed rest and regular neck collar protection were necessary. The operation was conducted only after anemia and hypoproteinemia had been effectively corrected, TB symptoms had been obviously relieved, and the erythrocyte sedimentation rate (ESR) had significantly declined.

### Operative procedure

All patients underwent tracheal intubation under general anesthesia. Patients were placed in a prone position for the procedure, and halo traction was maintained throughout to stabilize the spinal column. A posterior midline incision was made from the occipital protuberance to the C4 spinous process to expose the occipital bone, posterior arch of the atlas, and the C2–C4 spinous processes and vertebral laminae. Lateral mass screws were inserted in the C2–C4 segments, according to the extent of the lesion. Two pre-bent titanium plate-rods or titanium rods were installed. Six holes were drilled into the occipital bone through the orifices along the sides of the titanium plate (to avoid piercing the inner plate), and skull screws were inserted. After stable fixation had been achieved, hemostasis and irrigation were performed repeatedly. The bone graft bed was roughened, and autologous iliac bone or allogeneic bone was grafted.

Afterwards, the halo traction was removed, and the patients were placed in a supine position with slight cervical extension. Next, a left- or right-sided anterior cervical transverse skin incision was made. The subcutaneous tissue and platysma were stripped in layers. The superficial fascia was bluntly dissected along the anterior border of the sternocleidomastoid muscle, which was in line with the skin incision. Sharp dissection was carried out along the deep platysma to reveal the deep fascia, which was longitudinally incised along the inner edge of the sternocleidomastoid. Next, the supraomohyoid was found and incised. The prevertebral space was entered via the interspace between the internal edge of the vascular sheath and the outer edge of the visceral sheath. The prevertebral loose tissue was sharply dissected. Then, the intervertebral space of C2–C3 was located with the C-arm X-ray machine.

Important anatomical structures were protected during the surgery. The carotid artery and sternocleidomastoid were mobilized laterally, and the esophagus, trachea, and suprahyoid muscles were retracted medially. The superior thyroid vessels and hypoglossal nerve were identified and adequately protected. Only then did the surgeon introduce the endoscope and manipulating instruments such as curette and burr. After the endoscope was inserted, the posterior pharyngeal wall was dissociated, and the anterior arch of C1, the vertebral body of C2, and intervertebral space of C2–C3 were revealed under endoscopy. The sequestrum, abscesses, and other lesions were debrided. The abscess cavity was repeatedly washed. The surgical cavity was filled with absorbable gelatin sponge for hemostasis, and 1.0 g streptomycin and 0.3 g isoniazid were locally administered in the surgical field. Finally, a drainage tube was inserted, and the wound was closed in layers. An intraoperative biopsy specimen was subjected to mycobacterial culture and histopathological examination, and typical caseating granulomas were observed in all patients.

**Fig. 1:**
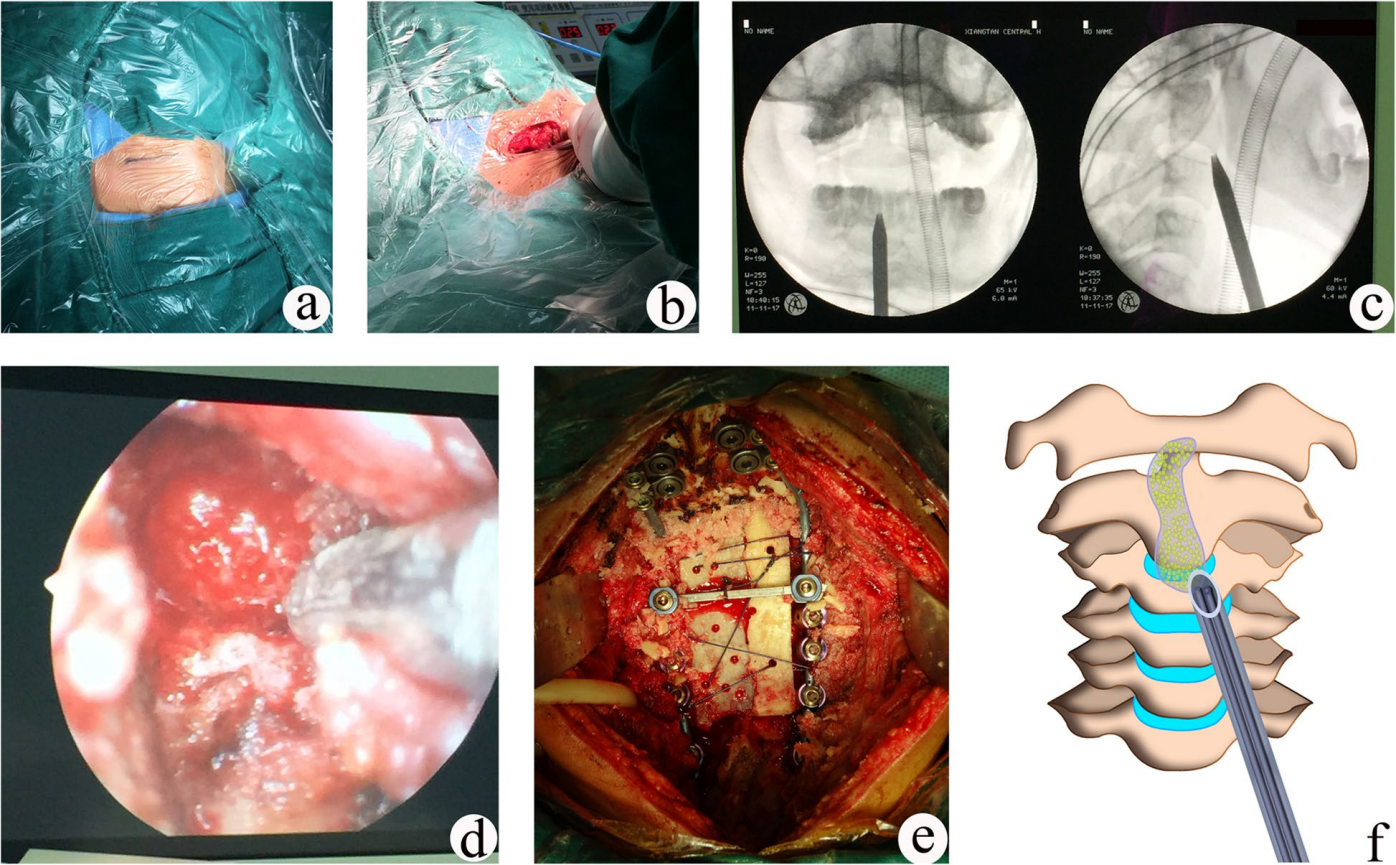
Schematic diagram of operative procedures were shown. **a** The patient was in a supine position with slight cervical extension. **b** Anterior cervical transverse skin incision was made. **c** Intraoperative positioning was performed with a C-arm machine. **d** The lesion was removed endoscopically. **e** Posterior fixation and bone grafting. **f** Schematic showing the use of the tubular retractor, which is placed against the anterior cervical spine via an anterolateral approach, and serves as a working channel for instruments and an endoscope

For patients with nerve compression, anterior endoscopic debridement was performed first, and then posterior fixation and bone grafting were performed, to avoid further aggravation of the spinal cord compression caused by lesions when repositioning the patient. The surgical procedure was depicted in Fig. 1.

### Postoperative care

When the drainage flow was < 30 mL/24 h, the drainage tube was removed. Intravenous antibiotics were used to prevent infection, and regular nutritional support was provided. After 5–7 days of bed rest, patients were allowed to ambulate while wearing a rigid cervical collar for effective neck support. The cervical collar was used for 3–6 months, and was removed when bone graft union was confirmed on X-ray examination. Depending on their general clinical condition, each patient was encouraged to undergo early physical rehabilitation to prevent thrombosis and improve nerve function. Standard anti-TB chemotherapy, as mentioned previously, was administered for 3 months, followed by 9–15 months of treatment with 450 mg/day rifampicin, 300 mg/day isoniazid, and 750 mg/day ethambutol.

### Follow-up evaluations

The extent of postoperative decompression and graft and instrumentation placement as well as graft fusion status were routinely assessed using X-ray or CT examination. Blood routine examination, and liver- and kidney-function tests were regularly conducted during the anti-TB treatment to detect any adverse drug reactions. The following factors were recorded preoperatively, immediately postoperatively, and during follow-up: (i) ESR, (ii) VAS score for neck pain, (iii) functional outcomes as assessed using the Kirkaldy–Willis criteria [11] and (iv) neurological status as assessed using the ASIA grade and JOA score,

### Statistical analysis

All statistical analyses were conducted using the SPSS version 24.0 statistical software package. The preoperative, postoperative, and follow-up clinical data were compared using the paired *t* test. Discrepancies in normal data distributions were analyzed using the rank sum test. *P* < 0.05 was considered to indicate statistically significant differences.

## Results

### Preoperative clinical information

The clinical data of the study patients are shown in Table 1. Of the 17 patients, 10 were men, and 7 were women. The average age of the patients was 36.8 ± 9.7 years, and the average duration of disease was 6.9 ± 2.4 months. The preoperative X-ray, CT, and MRI examinations showed blurring of the vertebral edges and bone destruction, retropharyngeal abscesses of different sizes, and soft-tissue swelling of the retropharyngeal wall in all patients. Symptomatic dysphagia occurred in none of the patients. In all, 8 patients had C1–2 spinal TB, 5 patients had C2 spinal TB, and 4 patients had C2–3 spinal TB.

**Table 1:** Clinical and demographic data of all patients

Patient no.	Gender	Age (years)	Affected level	Follow-up (month)	Operation time (min)	Blood loss (ml)	Disease duration (month)
1	M	18	C2-C3	72	180	300	10
2	M	28	C1-C2	63	210	400	4
3	F	46	C2-C3	63	200	350	11
4	F	40	C2-C3	66	230	400	7
5	M	32	C2	72	220	350	6
6	F	38	C2-C3	69	190	300	9
7	M	44	C1-C2	60	250	450	7
8	F	30	C2	60	240	430	3
9	M	24	C1-C2	75	200	320	5
10	M	54	C2	60	180	380	6
11	F	51	C2	63	220	400	8
12	M	35	C1-C2	78	200	450	9
13	M	42	C1-C2	66	180	400	10
14	F	40	C1-C2	72	220	300	4
15	M	35	C1-C2	81	230	350	5
16	F	26	C2	75	200	400	8
17	M	43	C1-C2	63	220	320	6
Mean		36.8 ± 9.7		68.1 ± 6.7	210.0 ± 21.2	364.7 ± 49.6	6.9 ± 2.4

*M* Male, *F* Female

### Postoperative data

**Fig. 2:**
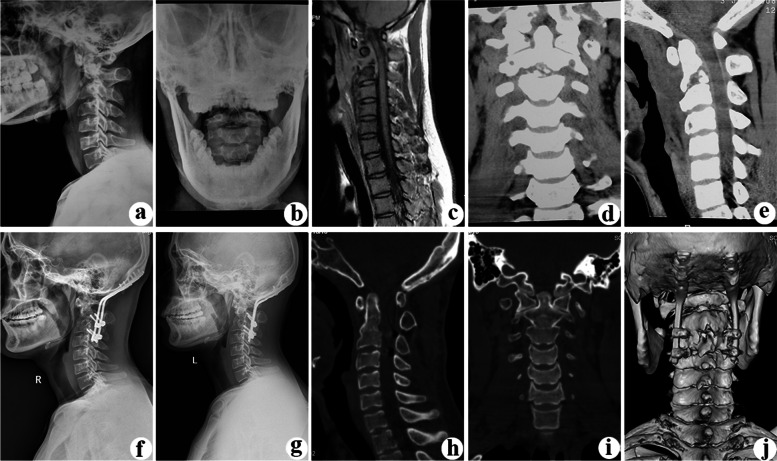
Endoscopy-assisted anterior cervical debridement combined with posterior fixation and fusion were performed for an 18-year-old man with upper cervical spine TB. Preoperative lateral (**a**) and open mouth (**b**) radiographs, MRI (**c**) and CT (**d**, **e**) show C2/3 vertebral destruction, spinal cord compression, and abscess formation. Postoperative X-ray examination (f) shows satisfactory posterior occipital-cervical fixation and a well-corrected cervical vertebral sequence. At the final follow-up at 72 months postoperatively, a radiograph (**g**) shows well-placed fixation, and CT (**h**–**j**) shows solid bony union

**Fig. 3:**
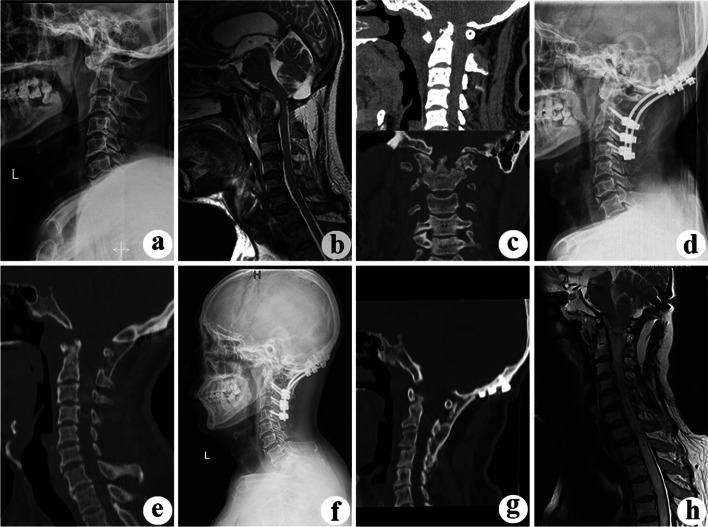
A 44-year-old man with upper cervical spine TB was treated using endoscopy-assisted anterior cervical debridement combined with posterior fixation and fusion. A preoperative lateral X-ray film (**a**), MRI (**b**), and CT (**c**) show C1/2 vertebral destruction, spinal cord compression, and abscess formation. A postoperative X-ray (**d**) shows satisfactory posterior occipital-cervical fixation and a well-corrected cervical vertebral sequence. CT (**e**) shows that the bone block is well placed. At the final follow-up at 60 months postoperatively, a radiograph (**f**) shows well-placed fixation, and CT (**g**) shows solid bony union. MRI (**h**) shows no abscess formation and no spinal cord compression

The mean operation time was 210.0 ± 21.2 min, and the mean blood loss was 364.7 ± 49.6 mL. The mean follow-up duration was 68.1 ± 6.7 months (Table 1). The ESR was elevated preoperatively (57.5 ± 12.9 mm/h) in all patients and returned to normal (9.6 ± 2.3 mm/h) by 3 months postoperatively (*P* < 0.05; Table 2). The symptoms of neck pain and stiffness were relieved after surgery in all patients. The average preoperative VAS score was 6.8 ± 0.7, which decreased to 1.6 ± 0.6 at 3 months postoperatively and to 0.2 ± 0.4 at the final follow-up (*P* < 0.05). Bone fusion was achieved in all patients by 10.9 ± 1.9 months after the surgery as determined using X-ray, CT, and MRI examinations (typical cases are shown in Figs. 2 and 3; Table 2). At the end of the follow-up period, according to the Kirkaldy–Willis criteria, 12 patients had excellent results, 4 patients had good results, and 1 patient had a fair result. None of the patients had a poor result (Table 2).

**Table 2:** Clinical effectiveness and outcomes

Patients no.	Fusion time (months)	ASIA		JOA		VAS			ESR (mm/h)		Kirkaldy-Willis criteria
		Pre	FFU	Pre	FFU	Pre	TMP	FFU	Pre	TMP	
1	9	E	E	15	17	7	2	0	54	9	Excellent
2	9	D	E	12	17	6	1	0	63	12	Excellent
3	12	C	E	9	16	7	2	0	58	11	Good
4	12	E	E	17	17	6	2	0	72	13	Excellent
5	15	D	E	13	17	8	2	1	47	9	Excellent
6	9	C	D	9	17	7	1	0	53	10	Excellent
7	9	D	E	12	16	6	1	0	38	6	Good
8	10	D	E	14	17	7	3	0	85	9	Excellent
9	9	E	E	16	17	7	1	0	56	10	Excellent
10	12	D	D	13	15	6	1	0	62	9	Fair
11	11	E	E	13	17	7	2	1	47	7	Excellent
12	12	D	E	12	17	7	2	1	51	10	Excellent
13	14	E	E	12	16	8	2	0	36	15	Excellent
14	9	D	E	13	16	7	1	0	63	7	Good
15	12	E	E	11	17	6	1	0	51	8	Excellent
16	12	E	E	10	16	6	1	0	66	8	Good
17	10	D	E	11	16	7	2	0	76	11	Excellent
Mean	10.9 ± 1.9			12.5 ± 2.2	16.5 ± 0.6^*^	6.8 ± 0.7	1.6 ± 0.6^*^	0.2 ± 0.4^*^	57.5 ± 12.9	9.6 ± 2.3^*^	

^*^ Analyzed by paired t test, compared with preoperative value, *P* < 0.05

*Pre* Pre-operative, *TMP* Three months post-operative, *FFU* Final follow-up

### Neurological outcomes

The neurological outcomes of the patients are presented in Table 2. Postoperative neurological deterioration did not occur in any patient. In all, 10 patients had incomplete paraplegia as a preoperative complication. Of these, 2 patients had grade-C disease, and 8 patients had grade-D disease according to the ASIA classification. At the final follow-up, the neurological status had returned to normal in 7 of the 8 patients with preoperative grade-D deficit, while 1 patient continued to have grade-D status. Among the 2 patients with grade-C status, 1 patient recovered to grade-E status, and the other recovered to grade-D status. The average JOA score significantly improved from 12.5 ± 2.2 preoperatively to 16.5 ± 0.6 at the time of the final follow-up examination (*P* < 0.05).

### Complications

All surgical wounds healed without chronic infection or sinus formation. No patient developed postoperative neurological deterioration. In addition, there were no complications related to instrumentation such as implant failure nor any radiographic signs of implant migration or loosening. Lastly, no other complications occurred such as dysphagia, dysphonia, or thromboembolism.

## Discussion

### Restoration of spinal stability

The objectives of the surgical treatment of upper spinal TB are radical debridement, decompression of the spinal canal, and restoration of spinal stability [12, 13]. Anterior debridement, internal fixation, and bone grafting are the conventional surgical methods for the treatment of the cervical spine [14]. However, in the absence of the vertebral body, there is no fixation space in the atlas. Currently, the common surgical methods for the treatment of upper cervical spine TB are focal debridement, bone graft fusion, and halo-vest external fixation [15]. However, external fixation does not fully meet the requirements of local stability, and can result in a low fusion rate, a high revision rate, and inconvenience in postoperative care.

Posterior cervical fixation and fusion are widely used for the restoration of cervical stability [16, 17]. Posterior upper cervical spine internal fixation can be achieved using steel wires, pedicle screws, lateral mass screws, and lamina hooks. Among these, lateral mass screws can not only be placed easily but also sufficiently stabilize the upper cervical spine [18–20]. Posterior occipital-cervical fusion can firmly stabilize the upper cervical spine while retaining the mobility of the lower cervical spine [21, 22].

In our study, posterior fixation was achieved using lateral mass screws and bone grafting. We used lateral mass screws and titanium plate-rods to fix the facet joints, and were able to firmly stabilize the posterior column of the cervical vertebrae. In addition, the bone graft bed and adequate allogeneic bone or autologous iliac bone should be carefully prepared to achieve firm fusion.

### Lesion debridement

Debridement is necessary for spinal healing and the restoration of spinal stability in patients with upper cervical spine TB [23–25]. The anterior approach is the best approach for surgical debridement to remove the anterior compression on the cervical spinal cord and cervical nerves caused by the vertebral destruction and posterior pharyngeal abscess resulting from upper cervical spine TB.

The transoral approach is conventionally used, but the surgical field is deep and narrow. In addition, this approach is associated with many complications, including mixed infection of the oral cavity, tongue edema, change in pronunciation/sound, posterior pharyngeal abscess, and cerebrospinal fluid leakage [26, 27]. Whitesides et al. [28] used the lateral retropharyngeal approach for the treatment of upper cervical spine TB and obtained good results. However, when the lesions were revealed using this approach, the carotid sheath was often incised, and it was difficult to remove the contralateral lesion because of obstruction by the ipsilateral vertebral artery; these factors may have a negative effect on the surgical outcomes [23]. Xing et al. [10] used anterior cervical debridement combined with posterior occipital-cervical fusion and fixation to treat upper cervical spine TB. However, the upper cervical spine, especially the atlantooccipital joint, is difficult to fully expose using this technique, owing to its high and deep anatomical position and the mandibular barrier. Thus, it is difficult to operate under direct vision due to the insufficient exposure and limited operation space, which increases the risk of iatrogenic damage to the esophagus, trachea, hypoglossal nerve, and blood vessels. Furthermore, as the deep part of the surgical field is often difficult to see under direct vision, radical focal debridement is difficult to achieve, and this may lead to a relapse.

Wolinsky et al. [9] used an endoscopic transcervical approach instead of the transpharyngeal approach to treat ventral compression of the brainstem and spinal cord in 3 patients, and achieved complete decompression in all patients. Moreover, none of the patients developed severe complications such as infection due to contamination with oral flora, meningitis, and poor pharyngeal healing. In addition, no patient required prolonged intubation, tracheostomy, or enteral tube feeding. In 2008, McGirt et al. [29] performed odontoidectomy using an endoscopic transcervical approach in 4 children with ventral lesions at the craniocervical junction; all children had satisfactory clinical outcomes at the final follow-up, and none required prolonged intubation or gastrostomy. Wang et al. [14] performed endoscopy-assisted anterior reconstruction and posterior fusion to treat ventral pathology in the upper cervical spine; successful clinical outcomes and radiographic fusion were documented in all patients at the final follow-up. The findings of the above studies show that endoscopy-assisted anterior transcervical decompression along with posterior fusion can result in satisfactory clinical and radiographic outcomes.

In this study, 17 patients with upper cervical spine TB underwent endoscopy-assisted anterior cervical debridement. The application of endoscopy has the advantages of a good light source and magnification. The lesion and local anatomical structure can be clearly displayed on the monitor. Moreover, it is easy to identify important tissues, which reduces the incidence of secondary injuries during the operation. In addition, the long handle of the endoscope allows operation in the deep field and can help comprehensively reveal the extent of the lesion, which would increase the chances of surgical success. Thus, the use of endoscopy can help minimize soft-tissue trauma by providing adequate visualization, making decompression of the upper cervical spine safer [30].

Unlike the endoscopic transnasal or transoral approaches, the endoscopic anterior transcervical approach provides a relatively wide surgical field in which decompression can be effective performed without causing significant splitting or retraction of tissues [31]. In addition, neurosurgeons and spine surgeons are familiar with the transcervical exposure. Finally, the postoperative recovery is faster, and patients can resume oral food intake sooner.

#### Limitations and precautions

The endoscopic surgical field is relatively limited. The surgeon must be familiar with the anatomical structure of the operative area or perform the operation with the assistance of experienced ENT doctors. The mode of endoscopic operation, namely, hand-eye matching, requires a certain period of adaptation.

During the operation, before the endoscope and manipulating instruments are inserted, important anatomical structures, such as the carotid artery, trachea, superior thyroid vessels, hypoglossal nerve, and in particular, the esophagus, need to be identified and protected. The strength and direction of the esophageal retractor should be controlled by grasping the retractor by the hand. The tail end of the retractor can be slightly turned up in order to press its front part against the surface of the vertebral body, which can prevent the esophagus from being inserted into the gap between the bottom of the tail end of the retractor and the vertebral body, and avoid injury. The strength applied on the esophageal retractor should be carefully controlled to prevent strain injury.

The operation should be very carefully performed when the lesion extends to the posterior edge of the vertebral body, as this is site is adjacent to the spinal cord and associated with a greater risk of bleeding. The aspirator head should be close to the bottom of the operating area to effectively remove the blood and keep the field clear and avoid stimulating the spinal cord. In addition, when continuous hemorrhage from the vertebral venous plexus occurs, the blood cannot always be removed in time, and it submerges the endoscope and obscures the surgical area. Double aspirators instead of a single aspirator with a large diameter can be used at the two ends of the operative area simultaneously, due to the limited surgical area. However, further study is required to improve aspirators and resolve the problem of endoscope submerging. Proper expansion of the original incision should be carried out to achieve good hemostasis and debridement when severe bleeding occurs or when the lesions are difficult to remove under endoscopy because of the limited surgical area.

However, radical debridement is relative and should be considered according to the degree of vertebral destruction; the blind extension of resection should be avoided. The purpose of debridement is to remove the lesion, including pus, caseous necrotic tissue, sequestrum, granulation tissue, necrotic intervertebral discs, and sclerotic bone, till the healthy or almost-healthy bone tissue is reached. In the case of stable sclerotic bone, radical debridement may not have to be performed, as this may result in spinal instability. Instead, only the hard and thick wall may be removed to improve the blood supply, and the residual lesion can be treated using drugs [32].

## Conclusion

Endoscopy-assisted anterior cervical debridement combined with posterior fixation and fusion is feasible and effective, and resulted in good clinical and radiographic outcomes in patients with upper cervical spine TB. However, the number of patients in this study was limited due to the rarity of upper cervical spine TB. Although all patients were followed up for a minimum of 5 years after the surgery, further studies with a large number of patients and long-term follow-up should be conducted.

## Data Availability

The datasets and materials generated or analyzed during the current study are available from the corresponding authors on reasonable request.

## Abbreviations

ASIA: American Spinal Injury Association
CT: computed tomography
ESR: erythrocyte sedimentation rate
JOA: Japanese Orthopaedic Association
MRI: magnetic resonance imaging
TB: tuberculosis
VAS: Visual Analogue Scale
